# Cost of diabetic retinopathy and macular oedema in a population, an eight year follow up

**DOI:** 10.1186/s12886-016-0318-x

**Published:** 2016-08-04

**Authors:** Pedro Romero-Aroca, Sofia de la Riva-Fernandez, Aida Valls-Mateu, Ramon Sagarra-Alamo, Antonio Moreno-Ribas, Nuria Soler, Domenec Puig

**Affiliations:** 1 Ophthalmology Service, University Hospital Sant Joan, Institut de Investigacio Sanitaria Pere Virgili [IISPV], University Rovira and Virgili, Reus, Spain; 2 Department of Computer Engineering and Mathematics, University Rovira and Virgili, Reus, Spain; 3 Health Care Area Reus-Priorat, Reus, Spain; 4 Institut Catala de la Salut [ICS], Barcelona, Spain; 5 Institut de Investigacio Sanitaria Pere Virgili [IISPV], University Rovira and Virgili, Reus, Spain; 6 Hospital Universtario Sant Joan, Avenida. Doctor. Josep Laporte 2, 43204 Reus, Spain

**Keywords:** Diabetic retinopathy, Diabetic macular oedema, Telemedicine, Cost of diabetic macular oedema, Cost of diabetic retinopathy screening

## Abstract

**Background:**

Prospective, population-based study of an 8-year follow up.

To determine the direct cost of diabetic retinopathy [DR], evaluating our screening programme and the cost of treating DR, focusing on diabetic macular oedema [DMO] after anti-vascular endothelial growth factor [anti-VEGF] treatment.

**Methods:**

A total of 15,396 diabetes mellitus [DM] patients were studied. We determined the cost-effectiveness of our screening programme against an annual programme by applying the Markov simulation model. We also compared the cost-effectiveness of anti-VEGF treatment to laser treatment for screened patients with DMO.

**Results:**

The cost of our 2.5-year screening programme was as follows: per patient with any-DR, €482.85 ± 35.14; per sight-threatening diabetic retinopathy [STDR] patient, €1528.26 ± 114.94; and €1826.98 ± 108.26 per DMO patient. Comparatively, an annual screening programme would result in increases as follows: 0.77 in QALY per patient with any-DR and 0.6 and 0.44 per patient with STDR or DMO, respectively, with an incremental cost-effective ratio [ICER] of €1096.88 for any-DR, €4571.2 for STDR and €7443.28 per DMO patient. Regarding diagnosis and treatment, the mean annual total cost per patient with DMO was €777.09 ± 49.45 for the laser treated group and €7153.62 ± 212.15 for the anti-VEGF group, with a QALY gain of 0.21, the yearly mean cost was €7153.62 ± 212.15 per patient, and the ICER was €30,361.

**Conclusions:**

Screening for diabetic retinopathy every 2.5 years is cost-effective, but should be adjusted to a patient's personal risk factors. Treatment with anti-VEGF for DMO has increased costs, but the cost-utility increases to 0.21 QALY per patient.

## Background

Diabetes mellitus [DM] is defined as a group of metabolic diseases whose common feature is an elevated blood glucose level [hyperglycaemia]. DM is a major health problem worldwide; by 2010 more than 200 million people had been diagnosed with diabetes and is predicted to increase by 62 % over the period 1995 to 2025 [1]. Diabetic retinopathy [DR] is DM microangiopathy in the retina, which is the most common cause of blindness in Europe [2]. To diagnose DR, the use non-mydriatic fundus retinography and telemedicine [3]. A screening programme obviously brings with it a cost to the public health system [4] that depends on the number of patients diagnosed with DR and the frequency of screening. The expansion of screening programmes that can diagnose patients with further ocular complications, such as diabetic macular oedema [DMO] has brought with it an increase in the overall cost of treatment. In the present study, we determine the direct cost of DR to our Health Care Area [HCA] between 2007 and 2014, including screening and the cost of diagnosis and treatment of those patients who had DR and DMO, focusing on the impact of that cost and the cost of the more recent anti-VEGF treatment.

## Methods

A population of 15,396, Caucasian, DM patients, which is 86.53 % [15,396/17,792] of all DM patients in our HCAs, were evaluated over an 8-year follow-up period. Our population is homologous with other areas in Spain.

### Study design

A prospective, population-based study, conducted from 1^st^ January 2007 to 31st December 2014. All diabetes patients screened annually within this period were included in the study.

The inclusion criteria were: patients with diabetes mellitus type 1 or 2 referred to our HCAs, the screening programme include the detection of DM patients by Family Physicians, who send the patients to non-mydriatic fundus camera unit, patients with HbA1c >7 %, insulin treatment or DM duration >10 years, are focused as high risk patients.

The exclusion criteria were: patients with other specific types of diabetes, those with gestational DM and patients with DR who live outside our HCA.

### Methods

We evaluated the cost of visits, examinations and interventions carried out for each patient during the 8-year follow-up. The classification used in the present study, taking into account an eye with a high level of DR, is: [i] no diabetic retinopathy [No-DR], [ii] any diabetic retinopathy [level 20 to 35 of ETDRS] [any-DR], [iii] sight-threatening diabetic retinopathy, defined as level 43 retinopathy or worse by the ETDRS [STDR], and/or [iv] sight-threatening maculopathy [STM]. DMO was classified as 'extrafoveal' or 'clinically significant [CSMO]' according to the ETDRS classification.

### Statistical methods

For the cost analysis, we used the Markov model with TreeAge Pro 6.0 statistical software. The sensitivity of our study was 90.2 % for DR, and the specificity was 98.6 %. Costs were standardized using data published by the Health Department of Catalonia [*CatSalut*] and the costs of pharmaceuticals, surgery material [5] and health staff spending of *Hospital Universitari Sant Joan*. In the present study we determined only direct costs. An annual 3 % discount rate was applied for future costs and utilities, consistent with the standard UK approach. The analysis was calculated in three steps: [i] a comparison of the cost of a 2.5-year screening programme with the cost of an annual programme, [ii] an evaluation of the cost of diagnosis and treatment of DR at the hospital, [iii] an analysis of the DMO treatment cost-utility, focusing on the inclusion of anti-VEGF drugs.

Our DR screening programme was evaluated by a cost-effectiveness study, comparing the current 2.5-year programme to a theoretical annual programme. Quality-adjusted life-years [QALYs] were used as the primary model outcome measure, using visual acuity as related time-trade off data for evaluating utility, and using life expectancy statistics for Spain in DM patients [78 years]. We also determined the incremental cost-effective ratio [ICER] in the analysis of annual costs against the cost of our current 2.5-year programme [6]. The rates of progression of DR is according to the meta-analysis of Wong et al., [7].

The cost of DMO [only CSMO type] treatment with anti-VEGF intravitreal drugs was evaluated by a cost-utility study based on the QALYs gained [8], considering the utility values most commonly accepted in the ophthalmology literature [8, 9]. We compare DMO patients treated with laser [2007 to 2010] and patients treated only by anti VEGF [2011 to 2014]. The comparison with patients treated with laser takes into account only the first eye treated. A descriptive statistical analysis of quantitative data was made. We used the analysis of frequency and percentage in each category. Differences between those included in the analyses were examined using the two-sample tailed *t*-test to compare two variables or a one-way ANOVA as if we were comparing more than two variables. Data evaluation and analysis was carried out using SPSS 22.0 statistical software package and *p* < 0.05 was considered statistically significant.

## Results

All 15,396 patients were screened, with a mean follow up of 3.18 ± 1.11 times for each patient over the 8 years. Screening took place every 2.5 years. The mean of the number of patients screened annual was 5507 ± 491.9 [5089, 6337], a mean percentage of 35.09 %. The whole sample included more males [m = 8168, f = 7227], which does in fact reflect the prevalence of diabetes in the population as a whole. The mean age of patients without DR was 65.66 ± 12.23 years, patients with any DR was 63.91 ± 11.85 years, patients with sight threatening diabetic retinopathy [STDR] was 61.48 ± 10.91 years and patients with DMO was 60.67 ± 10.37 years.

Table 1 shows the number of patients screened annually, and the incidence of DR at its different levels. The 8-year incidence of any-DR was 24.12 %, with an annual mean incidence value of 8.37 ± 2.19 % [8.09–8.99 %]. For STDR, the 8-year incidence was 7.59 % with an annual mean incidence value of 2.64 ± 0.15 % [2.48–2.88 %], and for DMO it was 6.36 % with an annual mean incidence value of 2.19 ± 0.18 % [2–2.49 %].

**Table 1:** Cases of diabetic retinopathy

	2007	2008	2009	2010	2011	2012	2013	2014
Patients screened	5027 32.65 %	4989 32.40 %	5312 34.50 %	5367 34.86 %	5276 34.22 %	6337 41.16 %	5623 36.52 %	6125 39.78 %
Annual incidence of any DR	407 8.09 %	402 8.06 %	428 8.06 %	432 8.05 %	426 8.07 %	556 8.77 %	502 8.92 %	551 8.99 %
Mild-DR	349 6.94 %	344 6.89 %	368 6.92 %	377 7.02 %	363 6.88 %	441 6,96 %	397 7.06 %	432
Moderate-DR	33 0.65 %	32 0.64 %	33 0.62 %	31 0.58 %	33 0.62 %	64 1 %	67 1.14 %	7.05 %
Severe-DR	24 0.48 %	26 0.52 %	26 0.49 %	24 0.48 %	30 0.57 %	47 0.74 %	43 0.76 %	65
Proliferative-DR	1 0.02 %	0	1 0.02 %	0	0	4 0.06 %	5 0.08 %	1.06 %
Total-DR^a^	407 8.09 %	402 8.06 %	428 8.06 %	432 8.05 %	426 8.07 %	556 8.77 %	502 8.92 %	551 8.99 %
Sight-Threatening DR	131 2.6 %	125 2.5 %	132 2.48 %	134 2.49 %	141 2.67 %	170 2.68 %	162 2.88 %	174 2.84 %
Extrafoveal-DMO	39 [0.75 %]	35 [0.72 %]	43 [0.84 %]	37 [0.69 %]	38 [0.72 %]	58 [0.92 %]	48 [0.86 %]	39 [0.75 %]
CSME^c^	65 [1.25 %]	66 [1.25 %]	69 [1.27 %]	77 [1.43 %]	72 [1.36 %]	92 [1.44 %]	87 [1.54 %]	65 [1.25 %]
Total-DMO^b^	104 2.00 %	101 2.02 %	112 2.11 %	114 2.12 %	110 2.08 %	150 2.36 %	135 2.40 %	153 2.49 %

DR^a^ diabetic retinopathy, DMO^b^ diabetic macular oedema, CSMO^c^ clinical significant macular oedema

### Analysis of cost of DR screening

The cost of screening remained stable from 2007 to 2014 [Table 2]. The mean cost was € 223,568 ± 20,956 [€201,840 to € 258,480]. Statistical analysis using the two-sample tailed *t*-test showed the differences in the total cost of screening were not significant [*p* = 0298; 95 % CI 201,802–237,717]. The annual mean screening cost per patient was € 40.53 ± 1.21. For a patient with any-DR the mean annual cost was € 482.85 ± 35.14; the annual mean cost of screening for a patient with STDR was € 1528.26 ± 114.94; and the mean annual cost for a patient with only DMO was € 1826.98 ± 108.26.

**Table 2:** Direct cost of screening of patients with DR

Year	2007	2008	2009	2010	2011	2012	2013	2014	Significance
Number of screened patients	5027 [32.65 %]	4989 [32.40 %]	5312 [34.50 %]	5367 [34.86 %]	5276 [34.22 %]	6337 [41.16 %]	5623 [36.52 %]	6125 [39.78 %]	NA^a^
Total cost of screening	€203,240	€201,840	€214,640	€217,080	€213,480	€258,480	€229,560	€250,224	*p* = 0,298
Mean cost of screening per patient	€40.43 ± 1.22	€40.45 ± 1.19	€40.40 ± 1.21	€40.44 ± 1.18	€40.46 ± 1.20	€40.43 ± 1.23	€40.42 ± 1.21	€40.45 ± 1.23	*P* = 0,186

NA^a^ non determined

Table 3 shows the cost of screening annually compared with the current programme. For a patient with any DR, the cost would be €482.32 with a QALY of 11.28. Screening every year would result in an increase of 0.77 in the annual QALY but at a cost of €1347.89. For a patient with STDR or DMO, the annual QALY would increase to values of 0.6 and 0.44 respectively. The ICER would increase the cost to €1096.88 for any-DR, € 4571.2 for STDR and € 7443.28 for DMO patients.

**Table 3:** Statistical analysis of cost of screening annually against the current 2.5 years

	Time of screening	Cost per patients diagnosed	QALY	ICER annual against 2.5 years	QALY increment
Any DR	1 year	€1347.89	12.05	€1096.88	0.77
	2.5 years	€482.32	11.28		
STDR	1 year	€4270.98	11.27	€4571.2	0.60
	2.5 years	€1528.26	10.67		
DMO	1 year	€5099.91	11.87	€7443.28	0.44
	2.5 years	€1824.82	11.43		

### Cost of DR diagnosis and treatment

The mean annual cost of diagnosis and treatment of patients with any-DR was €94,902 ± 19,576 [€77,995 to €128,359], with a mean cost per patient of €285.18 ± 21.61. Table 4 shows the cost of diagnosis and treatment of patients with DR by years, and Table 5 shows the analysis of the cost of diagnosis and treatment of patients with DR.

**Table 4:** Direct cost of diagnosis and treatment of patients with DR

Year	2007	2008	2009	2010	2011	2012	2013	2014	Significance
Total cost of diagnosis of DR patients	€62,347	€64,140	€63,822	63,702 €	€66,518	€67,164	€677,512	€667,717	*p* = 0.418
Total cost of DR diagnosis and treatment*	€81,262	€79,886	€82,334	€77,995	€85,598	€107,209	€116,574	€128,359	*P* < 0.001*
Mean total cost of diagnosis and treatment per patient	€199.66 ±19.92	€198.72 ±18.83	€192.37 ±20.77	€180.54 ±18.44	€200.93 ±20.32	€192.82 ±19.73	€232.22 ±19.64	€232.95 ±21.48	*p* = 0.007*

Total cost of DR diagnosis and treatment. *The significance is due to anti-VEGF treatment being included

**Table 5:** Direct cost of diagnosis and treatment per patient with DR

	Direct cost of screening programme to detect a patient	Direct cost of diagnosis per patient	Yearly direct cost of treatment per patient	Yearly direct cost of follow up per patient
Any DR	€482.32 ± 35.14	€100.51 ± 23.75	€184.67 ± 19.47	€288.15 ± 37.93
Sight threatening DR	€1528.26 ± 114.94	€167.91 ± 7.69	€2191 ± 15.51	€441.37 ± 10.83
DMO	€1824.82 ± 108.26	€132.24 ± 8.17	€777.09 ± 49.45^a^	€509.42 ± 20.28^a^
			€7153.62 ± 212.15^b^	€473.56 ± 62.69^b^
Mild DR	€443 ± 61.21	€79.45 ± 8.05	€139.27 ± 27.88	€145 ± 18.65
Moderate DR	€4995,93 ± 468,29	€108.12 ± 31.37	€5319.01 ± 1307.04	€448.76 ± 79.35
Severe DR	€7452,26 ± 698,53	€175.23 ± 48.21	€7970,93 ± 897.45	€475.13 ± 77.65
Proliferative	€94,332,48 ± 8842,19	€244.97 ± 87.16	€12177.74 ± 1027.22	€995.78 ± 97.47

^a^Treatment or follow up of DMO patients treated with laser, ^b^Treatment of DMO patients treated with anti-VEGF

### Analysis of cost-utility of diagnosis and treatment of DMO patients

Table 6 shows the total cost of patients with DMO over the 8 years. The group of patients treated by anti-VEGF drugs [2011 to 2014] was given an average of 5.7 injections the first year, 4.7 injections the second year and 2.9 the third year.

**Table 6:** Cost of diagnosis, treatment and follow up of diabetic macular oedema

Year	2007	2008	2009	2010	2011	2012	2013	2014
Number of patients with DMO	103	101	112	114	110	150	135	153
Cost of DMO diagnosis [Visit + FA + OCT]	€15,686	€15,382	€17,024	€17,328	€16,720	€16,752	€14,328	€16,238
Cost of grid laser treatment [Treatment + follow visits and OCT /FA] DMO treated with laser	€12,750	€11,250	€12,600	€13,050	No cases	No cases	No cases	No cases
Cost of 1 year follow up	€ 51,680	€ 48,120	€ 51,072	€ 52,896	No cases	No cases	No cases	No cases
Cost of Anti-VEGF treatment [Treatment + follow visits and OCT /FA] DMO Intravitreal Anti-VEGF	No cases	No cases	No cases	No cases	€389,327	€437,250	€472,929	€518,870
Cost of 1 year follow up	No cases	No cases	No cases	No cases	€39,510	€47,040	€48,048	€54,824
Cost of vitrectomy treatment [Treatment + follow visits and OCT /FA] DMO Vitrectomy	€5996	€3669	€5628	€6851	€4037	€6851	€3315	€8442
Cost of 1 year follow up	€2140	€1480	€2080	€2520	€1490	€2480	€1664	€3328
Treatment DMO cost	€56,880	€64,519	€71,380	€75,317	€459,384	€534,251	€561,956	€626,264
Total of the DMO cost	€72,566	€79,901	€88,404	€92,645	€476,104	€551,003	€576,284	€642,503
Mean cost of DMO per patient	€704.5 2 ± 34.23	€791.1 ±37.23	€789.32 ±41.99	€827.67 ±49.62	€4328.22 ±120.17	€3673.35 ±121.33	€4268.77 ±112.64	€4199.36 ±117.82

There were two groups of patients treated for CSMO:

- Group 1 treated by grid or focal laser [2007 to 2010] show a mean annual cost of €67,024 ± 8102€ [€56,880 to €75,317] or €777.09 ± 49.45 per patient.
- Group 2 treated by anti-VEGF intravitreal injections [2011 to 2014] show a mean annual cost of €545,464 ± 69,128 [€459,384 to € €626,264] or €7153.62 ± 212.15 per patient per year.

For group 1, the mean VA gain after laser treatment was 0.68 ± 1.78 [−11 to 6] letters, and the mean gain in VA for group 2 increased to 6.84 ± 3.22 [0 to 10] letters.

The mean cost per patient of total DMO treatment during a 3 year follow up, was €17791.99, which is from data only available for patients recruited between 2007 to 2013.

For the cost-utility analysis in DMO patients, we used the utility value according Brown et al. [8] for anti-VEGF drug differences with laser, and only the first year of treatment was taken into account. By mean age in DMO in the population [60.67 ± 10.37 years], we considered a further 17.33 years life expectancy in a Spanish population with DM [78 year], and the QALY gain value was 0.21. The yearly mean cost per patient was €7153.62 ± 212.15 per QALY, and the ICER was €30,361 per QALY.

## Discussion

In the present study, there are two key points to the cost of DR, the cost of screening and the cost of the DMO treatment, which has increased hugely since the introduction of anti-VEGF drugs. When screening for DR, retinography is the method recommended by scientific societies because it has proven to be cost effective [10, 11]. However, the present study shows that an 8.37 ± 2.19 % detection rate of DR per year [463 ± 63.61 patients] has a total cost of €223,568 ± 20,956. Furthermore, the annual percentage of patients with DR whose condition can lead to poor vision or blindness [STDR], is 2.64 ± 0.15 % [an average of 146.1 ± 19.45 patients] and DMO is detected in 2.19 ± 0.18 % of patients [122.4 ± 20.65 patients]. If these patients go on to develop poor vision or blindness it will represent a significant cost to society, so early detection is of great cost benefit [12].

Telemedicine screening for DR has been evaluated in different studies. Rein et al. in 2011 [13] demonstrated that a biannual eye examination by an ophthalmologist is more cost-effective than annual telemedicine. Examinations every 2 years might be cost-effective after one or more normal results and in a population with well-controlled type 2 diabetes [14]. There was essentially no risk of developing significant retinopathy within 3 years of a normal examination result [15]. The American Diabetes Association [2016] position statement concluded that if there is no evidence of DR in one or more eye examinations, then controls every 2 years can be considered adequate. If DR is present, subsequent examinations for patients with type 1 and type 2 DM should be repeated annually by an ophthalmologist or optometrist [16]. The present study demonstrates that for our population, a screening interval of 2.5 years is cost-effective, and annual screening would not improve the management of diabetes patients. For patients with STDR or DMO the results are not so conclusive, but these patients have poor vision, and therefore come for screening earlier than other patients with mild-DR. Furthermore, these patients have worse metabolic status, which requires more attention from their family doctor. It would seem sensible, therefore, to focus screening on patients whose characteristics can be defined as high-risk for developing DR, and we need to identify the patients who need more attention.

The present study also focused on the diagnosis and treatment of patients with diabetic retinopathy. Results show that treatment costs increased from €199.66 ± 19.92 in 2007 to €232.95 ± 21.48 in 2014. This increase can be explained by an increase in more severe forms of DR in recent years, related to poor metabolic control, as we described previously [17].

Finally, the study of DMO treatment costs was focused on the new intravitreal drugs. The introduction of anti-VEGF drugs for treatment of DMO [18] has been replacing laser treatment as the gold standard for DMO since 2011 according to ETDRS protocol [19] and for non-responsive DMO, we have recently been using corticoids, [20, 21]. In the present study, we only included anti-VEGF drugs because non-responsive cases appeared later than our study period. Table 6 shows the cost impact of DMO treatment by these drugs. It shows an exponential increase in the mean cost before 2011 of €67,024 ± 8102 to €545,464 ± 69,128 after 2011. Statistical analysis shows that anti-VEGF drugs are more effective than laser treatment for DMO patients, with an increase in visual acuity of 6.84 ± 3.22 letters in anti-VEGF treated patients against 0.86 ± 0.78 letters in the group treated with laser. This is an increase of 1.23 lines in ETDRS letters similar to the increase in VA observed in the READ 2 study, [22]. The QALY gain value was 0.21, a value similar to the 0.17 QALY gain in the Mitchell et al. study [23]. That study applied its model at 15 years from a baseline of 63 years whereas our model is based on 17 years with a mean patient age of 60 years. Applying the 15-year model to our results predicts a 0.18 QALY gain value, and for a younger population the anti-VEGF cost-effectiveness might be higher because of their longer rest-of-life expectancy.

The ICER of €30,361 per QALY gained relative to laser therapy is lower than the willingness-to-pay [WTP] that is required to be considered cost-effective by the National Institute for Health and Care Excellence [€38,460 or £30,000].

We can conclude, then, that treatment with anti-VEGF drug is cost-useful. Despite the present study being limited to the first year treatment follow up, the increase in VA remains stable, as found in other studies [24]. A difference worth noting is that in the first year the treatment, a mean of 5.7 injections of anti-VEGF per patient is fewer than in other studies such as RESTORE [23], with 10 injections the first year, or the RISE and RIDE clinical trials with 12 injections [25].

A potential weakness of our study is the use of medical record diagnoses and treatment from an integrated health care delivery system. The results of the present study do not represent all of the ophthalmology service costs of diabetes patients, because only patients referred from the screening programme have been evaluated rather than other patients from other hospitals. Weaknesses, also include the use of a screening programme can reduce the number of patients diagnosed with severe forms of DR, also the severity scale could vary if we use wide field image techniques, and the number of patients with STDR can increase. Finally, the number of patients who developed proliferative DR is small and can bias the statistical analysis.

The DMO treatment only takes into account the first treated eye, that which has a more advanced degree of oedema. In addition, the limitation of a 2-year follow up of each patient can reduce the total cost effectiveness for DMO. One strength is that it is a prospective, population-based study, representative of the Spanish population and representative of the standard management of patients with diabetic retinopathy, as opposed to studies that use the Markov or Monte Carlo models that simulate hypothetical cohorts [14, 26].

## Conclusion

A screening programme of 2.5 years by telemedicine is cost effective. The cost of screening can be reduced by better selection of time lapse individually tailored to each patient based on their personal risk factors. The cost of DMO increased after anti-VEGF drugs were introduced, but is cost-useful.
